# *Alteromonas nitratireducens* sp. nov., a Novel Nitrate-Reducing Bacterium Isolated from Marine Sediments, and the Evolution of Nitrate-Reducing Genes in the Genus *Alteromonas*

**DOI:** 10.3390/microorganisms13081888

**Published:** 2025-08-13

**Authors:** Ying-Li Chang, Jia-Xi Li, Xing-Chen Wang, Yang Li, Yun-Fei Cao, Xiang-Wen Duan, Cong Sun, Can Chen, Lin Xu

**Affiliations:** 1 College of Life Sciences and Medicine, Zhejiang Sci-Tech University, Hangzhou 310018, China; 2023220903001@mails.zstu.edu.cn (Y.-L.C.); 2022332871024@mails.zstu.edu.cn (J.-X.L.); 2023210901043@mails.zstu.edu.cn (Y.L.); 2023220902006@mails.zstu.edu.cn (Y.-F.C.); 15889305008@163.com (X.-W.D.); michael_sc@sina.com (C.S.); 2 Shaoxing Biomedical Research Institute of Zhejiang Sci-Tech University Co., Ltd., Zhejiang Engineering Research Center for the Development Technology of Medicinal and Edible Homologous Health Food, Shaoxing 312075, China; xc684107@outlook.com; 3 College of Life Sciences, Zhejiang University, Hangzhou 310058, China

**Keywords:** *Alteromonas nitratireducens* sp. nov., polyphasic taxonomy, comparative genomics, nitrate reduction, evolution

## Abstract

Nitrate reduction serves as a pivotal process in the global nitrogen cycle, playing a crucial role in natural ecosystems and industrial applications. Although the genus *Alteromonas* is not traditionally regarded as a nitrate reducer, several *Alteromonas* strains have recently been found to be capable of doing so. However, the evolutionary trajectory of this capability remains undiscovered. In this study, 32 bacterial strains were isolated and cultivated from the tidal flat sediment in Hangzhou Bay and classified into the classes *Cytophagia* (n = 2), *Alphaproteobacteria* (n = 2), *Gammaproteobacteria* (n = 17), *Flavobacteriia* (n = 5), and *Bacilli* (n = 6). One nitrate-reducing strain, designated as CYL-A6^T^, was identified by polyphasic taxonomy and proposed as a novel *Alteromonas* species. Genomic analysis reveals that seven *Alteromonas* genomes encode the dissimilatory nitrate reduction genes *narGHI*. Evolutionary analysis showed that these three nitrate-reducing genes were present in the early common ancestor of the genus *Alteromonas*, while gene loss events occurred in the subsequent evolution. With the loss of nitrate-reducing genes in the ancestry nodes, a wide variety of genes related to energy production and conversion, as well as carbohydrate, nucleotide, coenzyme, and inorganic ion metabolism, were gained in those nodes, which enabled *Alteromonas* members to utilize diverse substrates for increased energy production. This study enhances the understanding of microbial diversity in marine tidal flat sediments, proposes a novel nitrate-reducing species of the genus *Alteromonas*, and highlights the ecological diversification and ecological niche breadth in the evolution of the microbial metabolic network.

## 1. Introduction

Nitrogen acts as an essential element for all living organisms and is an indispensable component of DNA, RNA, proteins, and a wide variety of critical biological compounds [1,2]. Diverse marine prokaryotes are major drivers in the global nitrogen cycle, including ammonia assimilation, nitrogen fixation, nitrate reduction, nitrite reduction, denitrification, nitrification, and the conversion of ammonium to hydroxylamine [3,4]. Among these nitrogen pathways, nitrate reduction plays a pivotal role in promoting microbial growth and facilitating nitrogen reduction, highlighting its biological and ecological significance [4,5,6,7]. Nitrate reduction is commonly classified into two categories including assimilatory and dissimilatory ones. Nitrate is used as a substrate for biomass accumulations in assimilatory nitrate reduction, and as an electron acceptor for respiration in dissimilatory nitrate reduction [8,9]. As key players in nitrogen removal [10,11,12], nitrate-reducing bacteria have gained significant scientific interests worldwide.

The genus *Alteromonas* consists of 36 officially published species as of June 2025 (https://lpsn.dsmz.de/genus/alteromonas, accessed on 30 June 2025) [13]. *Alteromonas* bacteria are globally distributed in diverse marine waterbody and sediments, surviving from the coast to the deep sea [14,15,16,17,18]. *Alteromonas* members are ecologically active in marine environments and frequently interact with marine eukaryotes, driving the carbon, nitrogen, and iron biochemical cycles [19,20,21,22,23,24]. Although the genus *Alteromonas* is traditionally not regarded as a nitrate reducer [25], several *Alteromonas* strains have recently been found to be capable of doing so [18,26,27,28]. Genomic investigations reveal that an assimilatory nitrate reductase operon is located in the genomes of *A*. *macleodii* ATCC 27126^T^ [24], while a dissimilatory nitrate reductase operon is located in the ones of *A*. *facilis* P0213^T^ and ‘*A*. *arenosi*’ ASW11-36^T^ [27,28]. However, the evolutionary trajectory of the nitrate reduction operon is still unclear, hampering our understanding of the genetic and adaptation mechanisms of the genus *Alteromonas*. In this study, we isolated and cultivated a nitrate-reducing *Alteromonas* bacterium, designated as CYL-A6^T^, whose taxonomic status was determined using a polyphasic taxonomic approach. Furthermore, we performed comprehensive genomic and evolutionary investigations of high-quality *Alteromonas* genomes to elucidate the evolutionary trajectory of the nitrate reduction operon.

## 2. Materials and Methods

### 2.1. Strain Isolation, Cultivation, and Preservation

One tidal flat sediment sample was collected in Hangzhou Bay, PR China (121°58′54″ E, 29°16′17″ N) in March 2023 and stored at 4 °C until use. Approximately one gram of the sediment sample was resuspended in a sterile NaCl solution (3.0%, *w*/*v*), and the diluted samples were prepared using a ten-fold serial dilution method. Then, 200 μL of the subsample was plated onto marine agar 2216 (MA; Difco™, Becton, Dickinson and Company, Sparks, MD, USA) and incubated at 30 °C. After three days of cultivation, each single colony was picked and purified using repeated streaking to confirm the uniformity of colonial morphology.

Their taxonomic status was preliminarily determined using 16S rRNA gene sequence identity analysis as described by Wang et al. [29]. The genomic template DNA of each strain was extracted using a TIANamp bacterial DNA kit (Tiangen Biotechnology Co., Ltd., Beijing, China). Their 16S rRNA genes were amplified using the universal primer pair 27F (5′-AGAGTTTGATCCTGGCTCAG-3′) and 1492R (5′-GGYTACCTTGTTACTT-3′), and sequenced at Tsingke Biotechnology Co., Ltd. (Hangzhou, China). The 16S rRNA gene sequence identities of each strain compared with type strains were analyzed using the EzBioCloud web server (www.ezbiocloud.net/identify, accessed on 9 December 2024) [30]. Each strain was preserved in a glycerol solution (30%, *v*/*v*) at −80 °C for long-term preservation. Among those strains, one white colony, designated as CYL-A6^T^ and belonging to the genus *Alteromonas*, was selected to be subjected to a polyphasic taxonomic approach due to its low 16S rRNA gene sequence identities of ≤97.7% with *Alteromonas* type strains.

### 2.2. Phylogenetic Reconstruction and Genomic Analysis

#### 2.2.1. Phylogenetic Reconstruction Based on 16S rRNA Gene Sequences

The 16S rRNA genes of related *Alteromonas* type strains and the outgroup *Escherichia coli* NBRC 102203^T^ (accession number: AB681728) were obtained from the NCBI GenBank database. All 16S rRNA gene sequences were aligned with Clustal W version 2.0 [31] implemented in Molecular Evolutionary Genetics Analysis (MEGA) software version 11 [32]. Two phylogenetic trees, including neighbor-joining [33] and maximum-likelihood [34], were reconstructed using MEGA version 11 [32], Kimura’s two-parameter nucleotide substitution model [35] and a bootstrap resampling method with 1000 replicates. Phylogenetic trees were visualized using Microsoft^®^ PowerPoint^®^ 2019 version 2504.

#### 2.2.2. Genomic Sequencing, Assembly, and Annotation

Cells of strain CYL-A6^T^ were harvested by centrifuging at 12,000× *g* rpm for one minute, after the growth in marine broth 2216 (MB; Difco™, Becton, Dickinson and Company, Sparks, MD, USA) for three days at 35 °C. Genomic DNA was extracted from the cell pellets using an E.Z.N.A.^®^ Bacterial DNA Kit (Omega Bio-tek, Inc., Norcross, GA, USA) according to the manufacturer’s instructions. Sequencing libraries were prepared using an NEB Next^®^ Ultra™ DNA Library Prep Kit for Illumina^®^ (New England Biolabs, Ipswich, MA, USA) and then sequenced using the Illumina NovaSeq 6000 platform at Guangdong Magigene Biotechnology Co., Ltd. (Guangzhou, China) to generate 150 bp paired-end reads. Then, a draft genome was assembled using SPAdes version 3.10.1 [36] based on clean reads, which were filtered from raw reads by quality trimming. Other *Alteromonas* type strain genomes used in this study were obtained from the NCBI assembly database, with detailed information listed in Table 1.

Genome quality estimations were performed using CheckM version 1.2.2 [38] with the standard workflow. Genes including rRNA genes, tRNA genes, and open reading frames (ORFs) were predicted using Prokka version 1.14.6 [39] with the command “-kingdom Bacteria -gcode 11”. Functional annotations against Clusters of Orthologous Groups of proteins (COG), Gene Ontology (GO), and Kyoto Encyclopedia of Genes and Genomes (KEGG) databases were carried out on eggNOG-mapper webserver version 2 (http://eggnog-mapper.embl.de/, accessed on 15 January 2025) [40], and ecological genes were searched out based on key gene lists proposed by Royo-Llonch et al. [41].

#### 2.2.3. Comparative Genomic Analysis and Phylogenomic Reconstruction

Average nucleotide identity (ANI) and in silico DNA–DNA hybridization (*is*DDH) values between strain CYL-A6^T^ and *Alteromonas* type strains were carried out using OrthoANI version 1.40 [42] and the Genome-to-Genome Distance Calculator web server version 3.0 (http://ggdc.dsmz.de/ggdc.php, accessed on 6 January 2025) [43] to calculate their genomic relatedness indices. Aligned amino acid sequences of all high-quality *Alteromonas* genomes and its outgroup genome (*Escherichia coli* ATCC 11775^T^, NCBI assembly accession number: GCA_003697165.2) were obtained using GTDB-tk version 2.5.4 [44] based on the bac120_r220 database. The best amino acid substitution model of those aligned sequences was inferred using IQ-TREE v.1.6.12 [45] with the command “-m MFP”. Then, the maximum-likelihood phylogenomic tree was reconstructed using IQ-TREE version 2.3.6 [45] with ultrafast bootstrap values of 1000 and the amino acid substation models set as LG+F+I+R7. Finally, the ML phylogenomic trees were visualized using MEGA version 11 [32] and Microsoft^®^ PowerPoint^®^ 2019 version 2504.

### 2.3. Determinations of Phenotypic Characteristics

#### 2.3.1. Determination of Biochemical Characteristics

The reference strain *A*. *halophila* KCTC 22164^T^ was obtained from the Korean Collection for Type Cultures, and chosen for parallel comparisons with strain CYL-A6^T^. Temperature ranges for growth were tested using incubation at 10–50 °C (5 °C per interval) as well as at 4, 37, and 42 °C. Growth at different pH values (pH 5.0–10.0, pH 0.5 per interval) was assessed by adding appropriate biological buffers, including 2-morpholinoethanesphonic acid for pH 5.0–5.5, 3-morpholinopropanessulfonic acid for pH 6.0–7.5, Tricine for pH 8.0–8.5, and 3-cyclohexylamino-2-hydroxy-1-propanesonic acid for pH 9.0–10.0. Growth in the NaCl concentration range of 0–13.0% (*w*/*v*, 0.5% per interval) was determined in sodium-free MB prepared as described by Zhang et al. [46].

Morphological characteristics of colonies were observed using the naked eye, and those of cells were determined using transmission electron microscopy (JEM-1400Flash HC, JEOL Ltd., Okinawa, Japan) after strain CYL-A6^T^ was incubated on MA for three days at 30 °C. The motility ability was detected in the semi-solid MB medium containing 0.5% (*w*/*v*) agar by observing whether cells grew diffusely. Gram staining was tested as described previously [47]. Oxidase activity was tested via oxidation of *p*-aminodimethylaniline oxalate (1.0%, *w*/*v*). Catalase activity was determined through bubble production in hydrogen peroxide solution (3.0%, *v*/*v*). Growth in an anaerobic environment was determined using an AnaroPack^TM^ system (Mitsubishi Gas Chemical Company, Inc., Tokyo, Japan) with nitrate (0.5%, *w*/*v*) and nitrite (0.1%, *w*/*v*) added as electron acceptors. Hydrolysis determinations of casein; cellulose; starch; Tween 20, 40, 60, and 80; and tyrosine were performed as described by Xu et al. [48]. API 20NE, API ZYM, and API 50CH strips (bioMérieux Inc., Marcy-l’Étoile, France) were utilized for the determination of their biochemical, enzymatic, and acid production activities, according to the instruction manuals. Sole carbon, nitrogen, and energy source utilizations were performed through incubation in nutrient-free MB supplemented with carbohydrates (2 g/L), organic acids (1 g/L), or amino acids (1 g/L) including D-arabinose, L-arabinose, D-cellobiose, D-fructose, fucoidan, L-fucose, D-galactose, glucose, D-maltose, D-mannitol, D-mannose, D-melezitose, raffinose, rhamnose, D-sorbitol, starch, D-trehalose, xylan, D-xylose, succinate, L-alanine, L-cysteine, L-glutamic acid, L-lysine, L-methionine, L-tyrosine, and L-valine. Phenotypic and biochemical determinations were performed in three replicates.

#### 2.3.2. Determination of Chemotaxonomic Characteristics

For chemotaxonomic determinations, including isoprenoid quinone and polar lipids, cells of strain CYL-A6^T^ were harvested at the end of exponential growth, following growth in MB for 24 h at 37 °C. After isoprenoid quinone was extracted from lyophilized cells with chloroform/methanol (2:1, *v*/*v*), the resulting extract was filtered in the dark, and the resulting filtrate was then evaporated to dryness at 35 °C using a rotary evaporator (RE-52AA, Yarong, Shanghai, China). The isoprenoid quinone resuspended with 1 mL of chloroform was separated on a GF254 silica gel plate (10 × 20 cm; Merck Millipore, Darmstadt, Germany) with n-hexane-diethyl ether (34:6, *v*/*v*), followed by HPLC-MS analysis (Agilent 1200, Agilent Technologies, Inc., Santa Clara, CA, USA; Thermo Finnigan LCQ DECA XP MAX mass spectrometer, Thermo Fisher Scientific Inc., Waltham, MA, USA). Polar lipids were extracted following the described procedure and were subjected to differentiation between different polar lipids on 60 F254 silica gel plates (10 × 10 cm; Merck Millipore, Darmstadt, Germany) using two-dimensional thin-layer chromatography with chloroform/methanol/water (13:5:0.8, *v*/*v*/*v*) and chloroform/methanol/acetic acid/water (16:2.4:3:0.8, *v*/*v*/*v*/*v*). Total lipids, amino lipids, glycolipids, and phospholipids were identified using staining with phosphomolybdic acid, ninhydrin, molybdenum blue, and α-naphthol and sulfuric acid, respectively [49]. For fatty acid determinations, cells of strain CYL-A6^T^ and its reference strain, *A*. *halophila* KCTC 22164^T^, growing in quadrant 3 were harvested, when single colonies of them appeared in quadrant 4 on MA at their optimal temperatures for growth. Fatty acid methyl esters (FAMEs) were prepared using saponification, methylation, extraction, and lye washing. Subsequently, they were subjected to gas chromatography (Agilent 8860, Agilent Technologies, Inc., Santa Clara, CA, USA) and analyzed using the Sherlock Microbial Identification System (MIDI) and standard MIS library generation software version 6.5.

### 2.4. Nitrate Reduction Test and Its Evolutionary Trajectory Analysis

Nitrate reduction activity was also determined using nitrate broth and a nitrate reduction kit (Qingdao Haibo Biotechnology Co., Ltd., Qingdao, China). A total of 467 *Alteromonas* genomes were obtained from the NCBI assembly database. High-quality *Alteromonas* genomes with completeness of >90.0%, contamination of <5.0%, and the presence of 23S, 16S, and 5S rRNA genes, as well as 18 tRNA genes, as proposed by Bowers et al. [50], were retained for following genomic and phylogenomic analyses (Table S1). Comparative genomics were performed to search orthologous groups of genes using OrthoFinder version 2.5.4 [51]. Based on previous functional annotations of KEGG database, KEGG ko numbers of assimilatory/dissimilatory nitrate reduction including K00367 (ferredoxin-nitrate reductase; *narB*), K10534 (nitrate reductase (NAD(P)H); *NR*), K00360 (assimilatory nitrate reductase electron transfer subunit; *nasB*), K00372 (assimilatory nitrate reductase catalytic subunit; *nasA*), K00370 (respiratory nitrate reductase alpha subunit; *narG*), K00371 (respiratory nitrate reductase beta subunit; *narH*), K00374 (respiratory nitrate reductase gamma subunit; *narI*), K02567 (dissimilatory nitrate reductase (cytochrome); *napA*), and K02568 (dissimilatory nitrate reductase (cytochrome), electron transfer subunit; *napB*) were used to identify nitrate-reducing genes. Nitrate-reducing genes were double checked by using BLAST+ software version 2.5.0 [52] with bacterial NarGHI protein sequences from Uniprot database (NarG: P09152, P85097, P42175; NarH: I3R9M8, P11349, P85098, P42176, Q83RN5; NarI: P11350, P42177). The maximum-likelihood phylogenomic tree inferring the phylogenetic relationship was constructed as described above in Section 2.2.3. The evolutionary analysis of nitrate-reducing genes along a phylogenomic tree was performed using COUNT software version 10.04 [53], with ancestral reconstruction based on posterior probabilities in a phylogenetic birth-and-death model.

## 3. Results

### 3.1. Taxonomic Identification of Cultivated Strains Based on the 16S rRNA Gene Sequences

**Figure 1:**
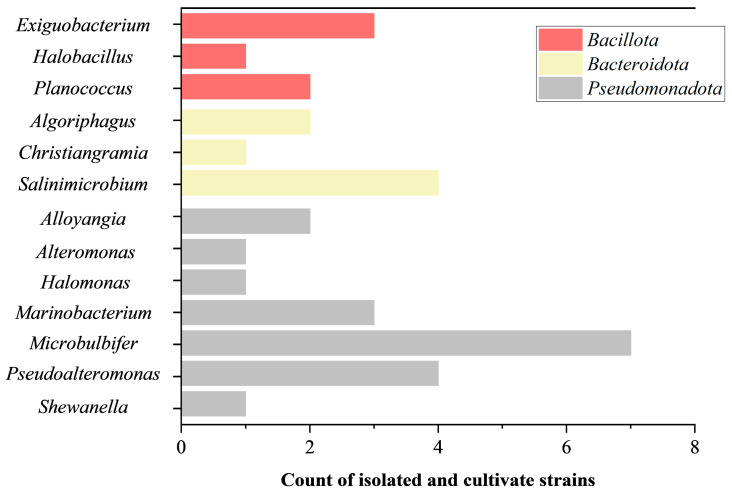
Genus classifications of 32 bacterial strains isolated and cultivated in this study. Red, yellow, and grey indicate the phyla *Bacillota*, *Bacteroidota*, and *Pseudomonadota*, respectively.

A total of 32 bacteria strains were successfully isolated and cultivated from this tidal flat sediment sample collected in Hangzhou Bay. The 16S rRNA gene sequence identity analysis pointed out that those strains could be classified into three phyla (Figure 1), including *Bacillota*, *Bacteroidota*, and *Pseudomonadota*. *Bacillota* strains were identified as three genera consisting of *Exiguobacterium* (n = 3), *Halobacillus* (n = 1), and *Planococcus* (n = 2). Bacteroidota strains were determined as three genera containing *Algoriphagus* (n = 2), *Christiangramia* (n = 1), and *Salinimicrobium* (n = 4). *Pseudomonadota* strains were regarded as seven genera comprising *Alloyangia* (n = 2), *Alteromonas* (n = 1), *Halomonas* (n = 1), *Marinobacterium* (n = 3), *Microbulbifer* (n = 7), *Pseudoalteromonas* (n = 4), and *Shewanella* (n = 1). Detailed 16S rRNA gene sequence identities and their top hit type strains are listed in Table S2. Among them, one white strain, designated as CYL-A6^T^ and belonging to the genus *Alteromonas*, was found to have low 16S rRNA gene sequence identities (≤97.7%) with existing type strains, indicating that strain CYL-A6^T^ was considered to represent a novel species in the genus *Alteromonas*. Meanwhile, strain CYL-A6^T^ was also deposited into the Korean Collection for Type Cultures (KCTC 8709^T^) and Marine Culture Collection of China (MCCC 1K09369^T^).

### 3.2. Polyphasic Taxonomy of Strain CYL-A6^T^

#### 3.2.1. 16S rRNA Gene Sequence Identity and Phylogenetic Relationship

Based on the 16S rRNA gene sequence identity comparisons, strain CYL-A6^T^ had the highest identity with *A*. *lipolytica* JW12^T^ and *A*. *aestuariivivens* JDTF-113^T^ (97.7%), followed by other *Alteromonas* type strains (<97.6%), indicating that those identities between strain CYL-A6^T^ and *Alteromonas* type strains were below the species threshold of 98.65% [54]. Therefore, strain CYL-A6^T^ could be regarded as a novel *Alteromonas* species based on its ribotype. Detailed 16S rRNA gene sequence identity results are listed in Table S3.

**Figure 2:**
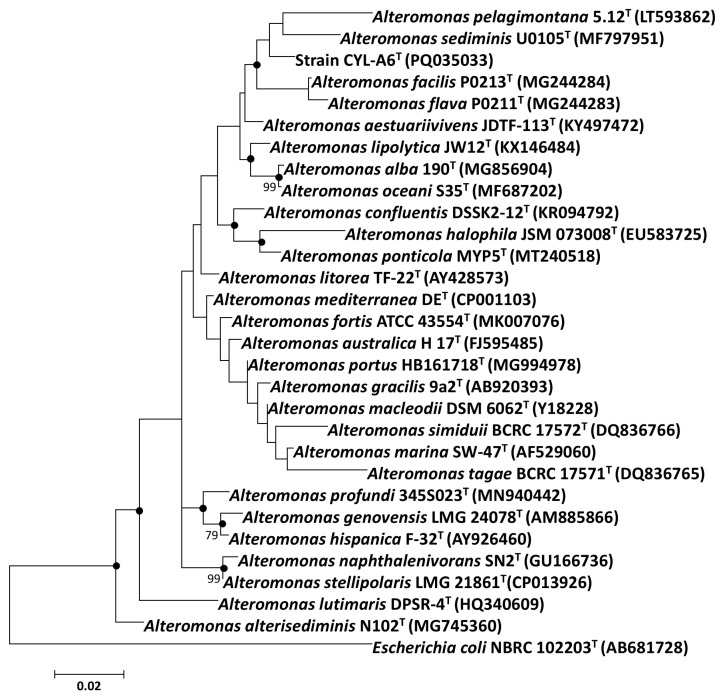
Maximum-likelihood phylogenetic tree based on 16S rRNA gene sequences showing phylogenetic relationships of strain CYL-A6^T^ and *Alteromonas* type strains. Bootstrap values are based on 1000 repetitions. Bar, 0.02 substitutions per nucleotide position. Filled circles indicate the same node recovered in the neighbor-joining phylogenetic tree (Figure S1). *Escherichia coli* NBRC 102203^T^ (AB681728) was used as an outgroup.

Both of the maximum-likelihood and neighbor-joining phylogenetic trees based on 16S rRNA gene sequences showed that strain CYL-A6^T^ was clustered into a clade consisting of *A*. *pelagimontana* 5.12^T^ and *A*. *sediminis* U0105^T^ (Figure 2 and Figure S1). However, their phylogenetic relationship was less supported with low bootstrap values of <70%. Therefore, the phylogenetic relationship of strain CYL-A6^T^ should be illustrated by the phylogenomic reconstruction, which contributes to the development of systematic taxonomies [55].

#### 3.2.2. Genomic Comparisons and Phylogenomic Relationship

The assembled genome of strain CYL-A6^T^ was composed of 48 contigs, with its genome size being 3,593,072 bp and a G+C content of 51.8%. Genomic coverage and the N50 value of this genome were 295× and 160,884 bp, respectively. The genomic completeness and contamination values of strain CYL-A6^T^ were 99.9% and 0.2%, respectively, indicating that its genome meets the criteria for high quality as proposed by Bowers et al. [50]. Gene predictions revealed that it contained four rRNA genes, 56 tRNA genes, and 3244 ORFs. Functional annotations against the COG, GO, and KEGG databases indicated that 2801 (86.3%, COG), 1112 (34.3%, GO), and 1932 (59.6%, KEGG) ORFs were assigned.

**Figure 3:**
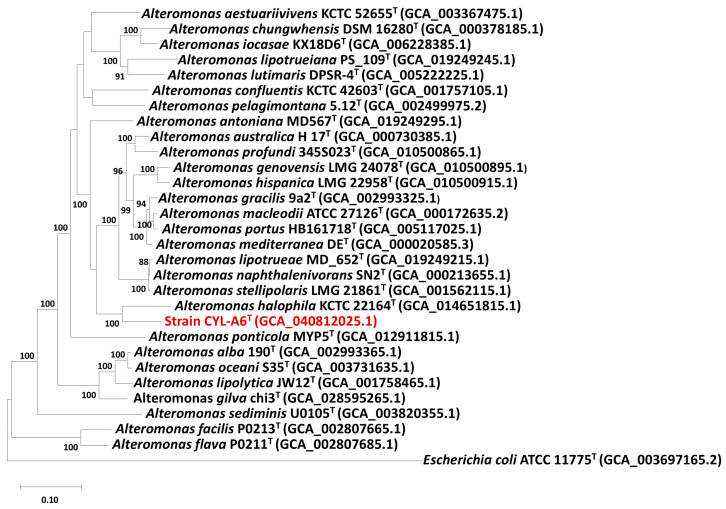
Maximum likelihood phylogenomic tree based on the GTDB database showing the phylogenetic relationships of strain CYL-A6^T^ (in red) and *Alteromonas* type strains. Bootstrap values are based on 1000 replicates. Bar, 0.1 substitutions per amino acid position. *Escherichia coli* ATCC 11775^T^ (GCA_003697165.2) was used as an outgroup.

Phylogenomic analysis based on the GTDB database revealed that strain CYL-A6^T^ and *A*. *halophila* KCTC 22164^T^ formed an independent clade, which was separated from other *Alteromonas* type strains (Figure 3). The ANI and *is*DDH values between strain CYL-A6^T^ and *Alteromonas halophila* KCTC 22164^T^ were 73.7% and 19.0%, respectively. Moreover, the ANI and *is*DDH values between strain CYL-A6^T^ and other *Alteromonas* species ranged from 69.0 to 73.7% and 18.8 to 23.8%, respectively (Table 2). These values fall below the species definition thresholds of ANI (95–96%) and *is*DDH (70%), supporting the classification of strain CYL-A6^T^ as a novel *Alteromonas* species [56].

Ecological gene annotations revealed that 21 ecological genes were encoded in the pan-genome of the genus *Alteromonas* (Table S1). The genome of strain CYL-A6^T^ encoded genes *accC* (acetyl-CoA carboxylase, biotin carboxylase subunit), *comA* (phosphosulfolactate synthase), *cysD* (sulfate adenylyltransferase subunit 2), *cysN* (sulfate adenylyltransferase subunit 1), *hoxH* (NAD-reducing hydrogenase large subunit), *ppc* (PEP carboxylase), and *prpE* (propionyl-CoA carboxylase), whose ecological gene distributions differed from those in the other 169 *Alteromonas* genomes (Table S1). Comparative genomics revealed that the genome of strain CYL-A6^T^ harbored 98 exclusive orthologous groups, which were absent in the other 169 *Alteromonas* genomes. Functional annotations revealed that 17 and 7 orthologous groups were assigned to COG and KEGG databases, respectively. COG annotations were related to categories C (energy production and conversion), H (coenzyme transport and metabolism), K (transcription), T (signal transduction mechanisms), O (posttranslational modification, protein turnover, chaperones), and S (function unknown), while KEGG annotations included amino acid *N*-acetyltransferase, erythromycin esterase, F-type H^+^-transporting ATPase subunit epsilon, putative membrane protein, thioesterase III, and uncharacterized protein (Table S4). Furthermore, three nitrate-reducing genes, including *narG* (respiratory nitrate reductase alpha chain), *narH* (respiratory nitrate reductase beta chain), and *narI* (respiratory nitrate reductase gamma chain) were found in the genome of strain CYL-A6^T^ (Table S5), demonstrating that strain CYL-A6^T^ could reduce nitrate to nitrite. However, these three genes were absent in the genome of *A*. *halophila* KCTC 22164^T^.

### 3.3. Phenotypic Characteristics of Strain CYL-A6^T^

**Figure 4:**
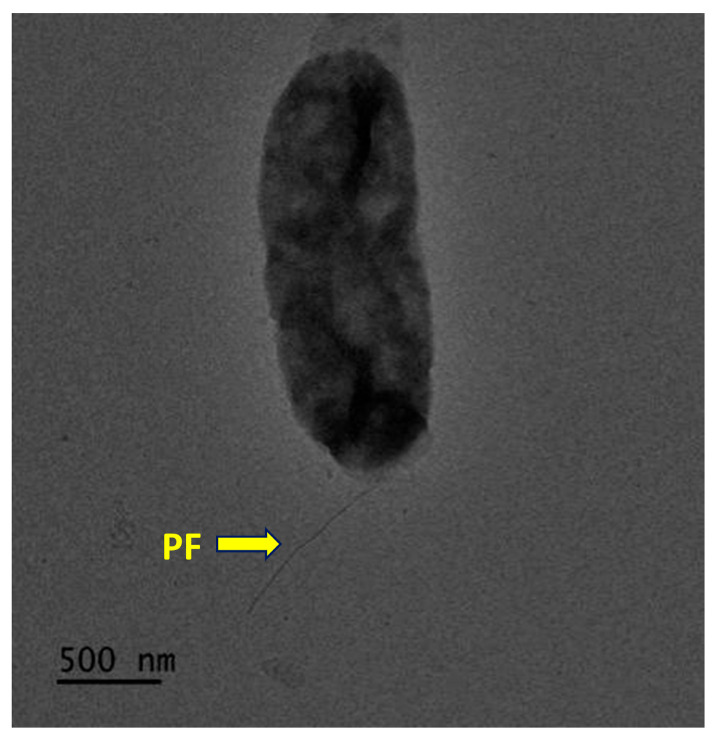
Transmission electron microscopic photographs of strain CYL-A6^T^ cells grown on MA for three days at 37 °C. PF indicates a single polar flagellum. Bar, 500 nm.

Strain CYL-A6^T^ grew at 20–45 °C, 1.0–12.0% (*w*/*v*) NaCl, and pH 5.5–9.0, with its optimal growth occurring at 37 °C, 3.5% (*w*/*v*) NaCl, and pH 7.0. Contrastingly, *A*. *halophila* KCTC 22164^T^ grew at 10–40 °C, 1.0–20.0% (*w*/*v*) NaCl, and pH 6.0–10.0, with its optimal growth occurring at 25–30 °C, 5.0–10.0% (*w*/*v*) NaCl, and pH 7.5. Cells of strain CYL-A6^T^ were Gram-negative, motile with a single polar flagellum, aerobic, and rod-shaped, measuring 1.5–1.8 and 0.5–0.7 μm in length and width, respectively (Figure 4). After incubation at 37 °C for 3 days, the colonies grown on MA were white and round, with flat and smooth edges and slightly raised centers with a diameter of 1.0–2.0 mm. Strain CYL-A6^T^ was positive for catalase and oxidase. It was similar to its reference strain, *A*. *halophila* KCTC 22164^T^, regarding hydrolytic activity, as neither hydrolyzed tyrosine, casein, starch, cellulose, or Tween 20, 60, and 80. In the API ZYM test, strain CYL-A6^T^ showed enzymatic activity identical to that of the reference strain across a wide range of enzymatic activities. Both were positive for acid and alkaline phosphatase, esterase (C4), esterase lipase (C8), lipase (C14), leucine and valine arylamidase, chymotrypsin, napthol-AS-BI-phosphopydrase, and α-glucosidase, and negative for trypsin, β-glucuronidase, β-glucosidase, *N*-acetyl-β-glucosaminidase, α-mannosidase, and β-fucosidase. Strain CYL-A6^T^ could be distinguished from *Alteromonas halophila* KCTC 22164^T^ by its β-glucosidase and α- and β-galactosidase activity. Moreover, strain CYL-A6^T^ could reduce nitrate, which was not reduced by *A*. *halophila* KCTC 22164^T^ (Figure S2). Furthermore, these two strains could be differentiated in several phenotypes including hydrolysis of Tween 40 and urea, acid production from D-glucose and D-mannose, and sole carbon source utilization of D-mannitol. Detailed differential phenotypic characteristics are shown in Table 3.

**Figure 5:**
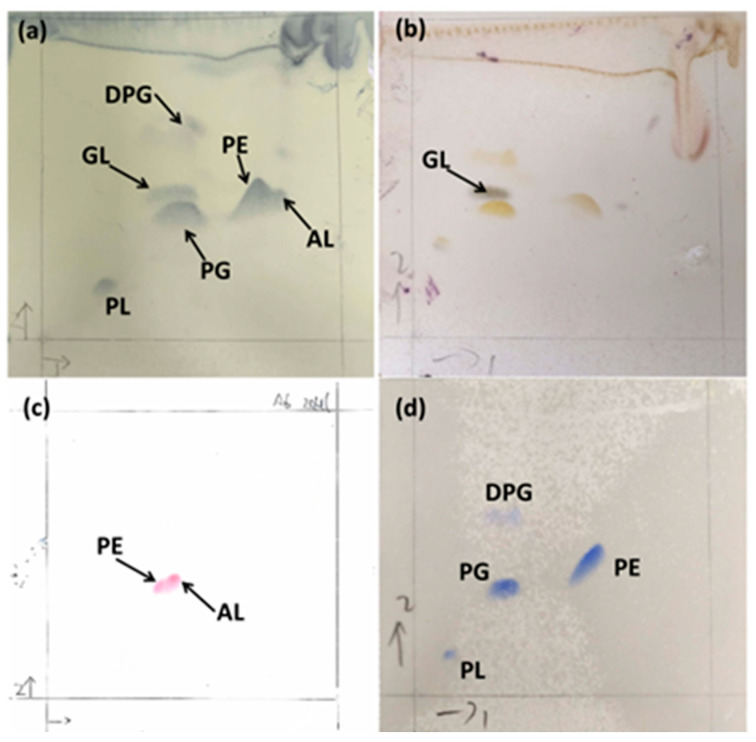
Polar lipid profiles including total lipids (**a**), glycolipids (**b**), aminolipids, (**c**) and phospholipids (**d**) of strain CYL-A6^T^. DPG, diphosphatidylglycerol; PE, phosphatidylethanolamine; PG, phosphatidylglycerol; AL, unidentified aminolipid; GL, unidentified glycolipid; PL, unidentified phospholipid.

The only respiratory quinone of strain CYL-A6^T^ was Q-8, which was consistent with the genus description of the genus *Alteromonas* [25]. Major fatty acids (>10%) of strain CYL-A6^T^ were C_16:0_, summed feature 3 (C_16:1_ *ω*6*c* and/or C_16:1_ *ω*7*c*), and summed feature 8 (C_18:1_ *ω*6*c* and/or C_18:1_ *ω*7*c*), which were similar to *A*. *halophila* KCTC 22164^T^ (Table 4). Furthermore, the polar lipid profile of strain CYL-A6^T^ contained diphosphatidylglycerol, phosphatidylethanolamine, phosphatidylglycerol, one unidentified aminolipid, one unidentified glycolipid, and one unidentified phospholipid (Figure 5), among which diphosphatidylglycerol and unidentified aminolipids and glycolipids were not detected in the polar lipid profile of *A*. *halophila* KCTC 22164^T^.

### 3.4. Evolutionary Trajectory of Nitrate-Reducing Genes in the Genus Alteromonas

Functional annotations revealed that nitrate-reducing genes *narGHI* were encoded in the seven *Alteromonas* genomes, including ones of strain CYL-A6^T^, *A*. *alba* 190^T^, ‘*A*. *arenosi*’ ASW11-36^T^, *A*. *facilis* P0213^T^, *A*. *mediterranea* 615, *A*. *mediterranea* DE^T^, and *A*. *mediterranea* CH_XMU1405-1 (Table S6). In addition, these five nitrate-reducing genes were clustered in those genomes with the same gene order: *narX*, *narG*, and *narH*. No other nitrate-reducing genes were found in the other *Alteromonas* genomes.

**Figure 6:**
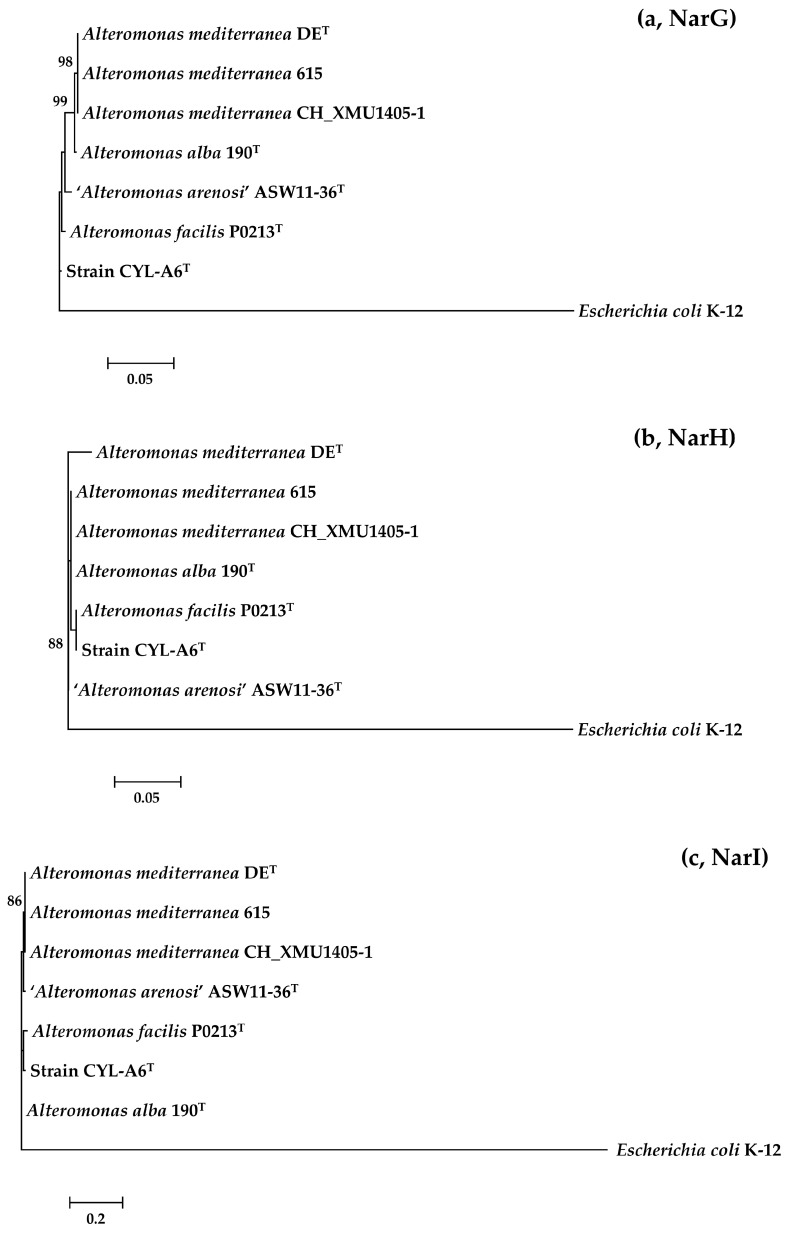
Maximum-likelihood phylogenetic trees based on the amino acid sequences of proteins NarG (**a**), NarH (**b**), and NarI (**c**). Based on the amino acid substitution model inferred using IQ-Tree version 1.6.12 [64], those used in the phylogenetic reconstructions were Q.pfam+G4 (NarG), WAG (NarH), and mtZOA+G4 (NarI), respectively. Bar, 0.05 (**a**,**b**) and 0.2 (**c**) substitutions per amino acid position.

To detect the possible horizontal gene transfer of nitrate-reducing genes, we performed a composition-based approach (including sequence identities) and a phylogeny-based approach (including phylogenetic relationships and evolutionary distances) to infer different origins [58]. Nucleotide sequence identities of genes *narGHI* in the *Alteromonas* members were 88.6–100.0% (*narG*), 89.5–99.9% (*narH*), and 87.3–100.0% (*narI*), and amino acid sequence identities of proteins NarGHI in them were 98.6–100.0% (NarG), 99.4–100.0% (NarH), and 97.4–100.0% (NarI), indicating that they shared high sequence homologies. Detailed pairwise nucleotide/amino acid sequence identities are shown in Table S7. Moreover, phylogenetic reconstructions based on the amino acid sequences also revealed low evolutionary divergence among the seven *Alteromonas* members, with maximum substitutions per amino acid position of 0.014 (NarG), 0.023 (NarH), and 0.037 (NarI) (Figure 6). Therefore, nitrate-reducing genes in the genus *Alteromonas* were not horizontally transferred. Comparative genomic analysis found that a total of 15 OCs were exclusively present in the *Alteromonas* genomes encoding nitrate-reducing genes (Table S8). Except for those three respiratory nitrate reductase genes *narGHI*, functional annotations based on the KEGG database revealed that the other twelve OCs included *narJ* (nitrate reductase molybdenum cofactor assembly chaperone, K00373), *narK* (nitrate/nitrite transporter, K02575), *narQ* (nitrate/nitrite sensor histidine kinase, K07674), *nrdD* (ribonucleoside-triphosphate reductase, K21636), *nrdG* (anaerobic ribonucleoside-triphosphate reductase activating protein, K04068), *ppiC* (peptidyl-prolyl cis-trans isomerase C, K03769), and five unannotated ones. Among them, genes *narJKQ* were related to nitrate reduction [59,60,61,62,63], and absent in other *Alteromonas* genomes, confirming their nitrate-reducing activity.

**Figure 7:**
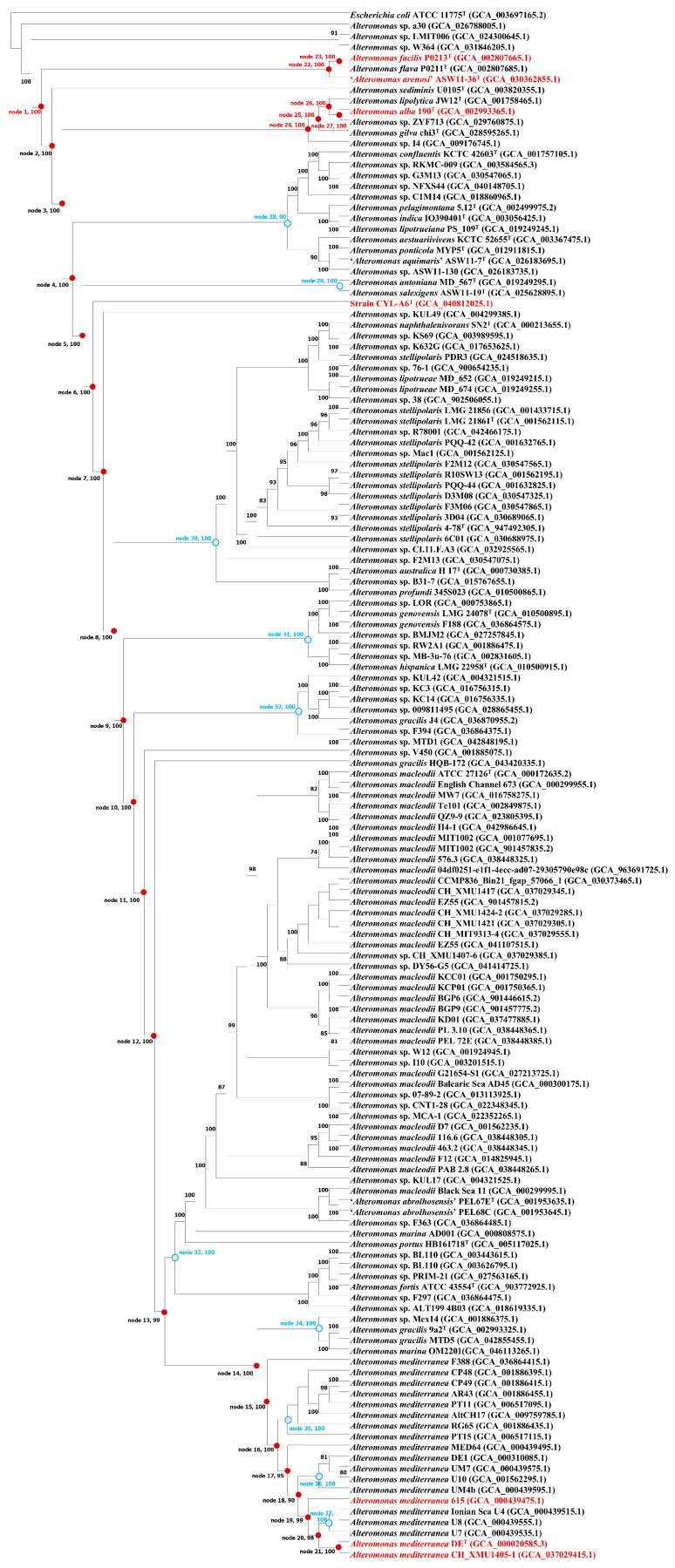
Evolutionary trajectory of nitrate-reducing genes in *Alteromonas* members and their ancestry nodes. *Alteromonas* members containing nitrate-reducing genes are shown in red. Filled red nodes represent ancestral nodes encoding nitrate-reducing genes, while blue and grey filled nodes show ancestral nodes not harboring nitrate-reducing genes.

Phylogenomic reconstruction revealed that nitrate-reducing genes were discretely distributed in the *Alteromonas* members (Figure 7). However, the evolutionary analysis of genes *narGHI* along the phylogenomic tree of the genus *Alteromonas* indicated that those genes were first present in the ancestor of all *Alteromonas* members excluding *Alteromonas* sp. a30, *Alteromonas* sp. LMIT006, and *Alteromonas* sp. W364 (node 1, Figure 7). Those genes were also found in the early ancestors of the genus *Alteromonas*, including nodes 2–27 (Figure 7). In contrast, nitrate-reducing genes *narGHIJX* were absent in most of the existing *Alteromonas* genomes, due to gene losses, especially in the ancestor nodes including nodes 28–37 (Figure 7). Moreover, ancestral genome reconstruction revealed that the genome of node 1 harbored 2469 genes classified into 2413 OCs, among which 210 OCs were gained in nodes 28–37 encoding 2601–3919 genes grouped into 2553–3690 OCs (Table S9). COG and KEGG annotations of those gained OCs showed that they were mostly related to metabolism, including energy production and conversion as well as amino acid, carbohydrate, and lipid transport and metabolism (Table 5).

## 4. Discussion

### 4.1. Proposal of Alteromonas nitratireducens sp. nov.

The 16S rRNA gene sequence identity analysis revealed that strain CYL-A6^T^ had low identities of ≤97.7% with *Alteromonas* type strains, demonstrating that strain CYL-A6^T^ could be differentiated from existing *Alteromonas* species based on the threshold of <98.65% of the 16S rRNA gene sequence identity for delineating two different species [54]. Overall genome relatedness indices including ANI and *is*DDH values between CYL-A6^T^ and *Alteromonas* type strains were also lower than the proposed species thresholds of 95.0–96.0% and 70.0%, respectively [56]. The maximum-likelihood phylogenomic tree based on the GTDB database showed that strain CYL-A6^T^ was solidly clustered in a clade with *A*. *halophila* KCTC 22164^T^ but separated from other *Alteromonas* type strains. Compared with *A*. *halophila* KCTC 22164^T^, strain CYL-A6^T^ could be differentiated regarding nitrate reduction, optimal NaCl concentration for growth, hydrolysis of Tween 40 and urea, α- and β-galactosidases and β-glucosidase activities, sole carbon source utilization of D-mannitol, acid production from D-glucose and D-mannose, the presence of diphosphatidylglycerol, an unidentified aminolipid, and an unidentified glycolipid. Based on those genetic, genomic, phylogenomic, biochemical, and chemotaxonomic characteristics, strain CYL-A6^T^ could be identified as a novel *Alteromonas* species, for which the name *Alteromonas nitratireducens* sp. nov. is proposed.

### 4.2. Description of Alteromonas nitratireducens sp. nov.

*Alteromonas nitratireducens* (ni.tra.ti.re.du’cens. N.L. masc. n. *nitras*, nitrate; L. pres. part. *reducens*, bringing back to a state or condition; N.L. fem. part. adj. *nitratireducens*, reducing nitrate).

Cells are Gram-negative, motile with single polar flagellum, aerobic, and rod-shaped, measuring 1.5–1.8 μm in length and 0.5–0.7 μm in width. Following cultivation for 3 days at 37 °C on MA, the colonies are white and round with a flat and smooth edge and a slightly raised center, measuring 1.0–2.0 mm in diameter. Growth occurs at 20–45 °C, 1.0–12.0% (*w*/*v*) NaCl, and pH 5.5–9.0, with optimal growth at 37 °C, 3.5% (*w*/*v*) NaCl, and pH 7.0. It is catalase- and oxidase-positive. It does not hydrolyze tyrosine, casein, starch, cellulose or Tween 20, 40, 60, and 80. In the API ZYM test, it is positive for acid and alkaline phosphatase, esterase (C4), esterase lipase (C8), lipase (C14), leucine and valine arylaminase, chymotrypsin, α-glucosidase, and naphthol-AS-BI-phosphohydrolase. It is negative for cystine arylaminase, trypsin, α- and β-galactosidase, β-glucuronidase, β-glucosidase, *N*-acetyl-β-glucosaminidase, and α- and β-mannosidase. In the API 20NE test, it is positive for nitrate reduction; hydrolysis of aesculin, gelatin, and urea; and assimilation of glucose, arabinose, mannose, mannitol, *N*-acetylglucosamine, maltose, gluconate, malic acid, and citric acid. It is negative for indole production, glucose fermentation, arginine dihydrolase and β-galactosidase activities, and assimilation of adipic acid, capric acid, and phenylacetic acid. Acid is produced from *N*-acetylglucosamine, D-arabinose, L-arabinose, esculin ferric citrate, D-fucose, D-galactose, D-glucose, glycerol, D-maltose, D-mannose, D-ribose, and D-xylose, while it is not produced from D-adonitol, amygdalin, D-arabitol, L-arabitol, arbutin, D-cellobiose, dulcitol, erythritol, D-fructose, L-fucose, gentiobiose, glycogen, inositol, inulin, D-lactose, D-lyxose, D-mannitol, D-melibiose, D-melezitose, methyl-α-D-glucopyranoside, methyl-α-D-mannopyranoside, methyl-β-D-xylopyranoside, potassium gluconate, potassium 2-ketogluconate, potassium 5-ketogluconate, D-raffinose, L-rhamnose, salicin, starch, D-sorbitol, L-sorbose, sucrose, D-tagatose, D-trehalose, D-turanose, xylitol, or L-xylose. D-cellobiose, glucose, D-mannose, D-melezitose, raffinose, D-sorbitol, D-trehalose, L-alanine, L-cysteine, L-lysine, L-tyrosine, and L-valine are utilized as the sole carbon, nitrogen, and energy sources, while D-arabinose, L-arabinose, fucoidan, L-fucose, D-fructose, D-galactose, D-maltose, D-mannitol, rhamnose, xylan, D-xylose, starch, succinate, L-glutamic acid, and L-methionine are not utilized as the sole carbon, nitrogen, and energy sources. The only respiratory quinone is Q-8. Major fatty acids (>10%) are C_16:0_, summed feature 3 (C_16:1_*ω*6*c* and/or C_16:1_*ω*7*c*), and summed feature 8 (C_18:1_*ω*6*c* and/or C_18:1_*ω*7*c*). The polar lipid profile contains diphosphatidylglycerol, phosphatidylethanolamine, phosphatidylglycerol, one unidentified aminolipid, one unidentified glycolipid, and one unidentified phospholipid.

The type strain is CYL-A6^T^ (= KCTC 8709^T^ = MCCC 1K09369^T^)_,_ which was isolated from marine sediments collected in Hangzhou Bay, China. The genomic DNA G+C content of the type strain is 51.8%. The NCBI GenBank accession numbers for the 16S rRNA gene and genome sequences of strain CYL-A6^T^ are PQ035033 and JBFNPK000000000, respectively.

### 4.3. Loss of Nitrate-Reducing Genes and Gain of Other Metabolism Genes

Nitrate reduction is classified into two categories, including assimilatory nitrate reduction utilizing nitrate as a substrate for biomass accumulation and dissimilatory nitrate reduction using nitrate as an electron acceptor for respiration [8,9]. Our genomic investigation revealed that seven *Alteromonas* members are capable of performing dissimilatory nitrate reduction, confirmed by the presence of genes *narGHI* encoding respiratory nitrate reductase and biochemical determination. Though those seven *Alteromonas* members are phylogenetically separated in different clades, our evolutionary analysis indicated that the most recent common ancestor of almost all *Alteromonas* members contains those three nitrate-reducing genes. Pairwise gene/protein sequence identities based on *narGHI*/NarGHI of seven *Alteromonas* members are high, and evolutionary distances based on their NARGHI phylogenies are close. Moreover, other nitrate metabolism genes including *narJKQ* are also only present in those seven *Alteromonas* members. These results demonstrate that gene loss is one of the major evolutionary forces driving the nitrate-reducing genes in the genus *Alteromonas*.

Dissimilatory nitrate reduction facilitates prokaryotes to obtain energy in both aerobic and anaerobic conditions [65,66], especially for those prokaryotes surviving in wetland sediments, estuarine sediments, and rice paddies, where the oxygen content is relatively low [67,68,69]. However, extensive loss of nitrate-reducing genes in the genus *Alteromonas* demonstrates this metabolic pathway tends to be dispensable in this genus. In the evolution of prokaryotes, extensive gene gain and loss events have altered their genomic contents [70]. With the loss of nitrate-reducing genes in the ancestry nodes, a wide variety of genes related to other metabolic pathways including fructose and mannose metabolism, galactose metabolism, glycolysis, methane metabolism, nitrogen metabolism, oxidative phosphorylation, purine metabolism, pyruvate metabolism, starch and sucrose metabolisms, and thiamine metabolism are gained in those nodes (Table 5). These metabolic pathways contribute to energy production, conversion, and transfer [71,72,73,74,75,76,77,78]. Therefore, most *Alteromonas* members have evolved to utilize diverse substrates for energy production, rather than relying on dissimilatory nitrate reduction to generate energy, highlighting their ecological diversification and broadening of ecological niche in the evolution of microbial metabolic networks.

## 5. Conclusions

In this study, 32 bacterial strains were isolated and cultivated from tidal flat sediment samples collected from Hangzhou Bay, and classified into five classes including *Cytophagia* (n = 2), *Alphaproteobacteria* (n = 2), *Gammaproteobacteria* (n = 17), *Flavobacteriia* (n = 5), and *Bacilli* (n = 6) based on 16S rRNA gene sequence identity analysis. Among those strains, one nitrate-reducing strain, designated as CYL-A6T, had low identities of <97.7%, as well as ANI and *is*DDH values of <95.0–96.0% and <70.0% with *Alteromonas* type strains. Compared with its reference strain, strain CYL-A6^T^ could be differentiated from others regarding several biochemical and physiological characteristics, as well as polar lipid profiles, indicating that strain CYL-A6^T^ could be identified as a novel *Alteromonas* species, for which the name “*Alteromonas nitratireducens* sp. nov.” is proposed. Comparative genomic analysis of *Alteromonas* genomes indicated that dissimilatory nitrate reduction genes *narGHI* were annotated in seven *Alteromonas* genomes. Evolutionary analysis showed that these three nitrate-reducing genes were present in the early common ancestor of the genus *Alteromonas*. The high sequence identity and close evolutionary distance of the *narGHI* genes in seven genomes revealed that gene loss was considered a driving force in the evolution of nitrate reduction genes in the genus *Alteromonas*. A wide variety of genes related to energy production and conversion, as well as carbohydrate, nucleotide, coenzyme, and inorganic ion metabolisms, were gained in other nodes that had lost *narGHI* genes. This enabled most *Alteromonas* members to evolve to utilize different substrates for energy production. This study enhances our understanding of microbial diversity in marine tidal flat sediments, proposes a novel nitrate-reducing species of the genus *Alteromonas*, and highlights the ecological diversification and niche breadth in the evolution of the microbial metabolic network.

## Figures and Tables

**Table 1:** Genomic information on *Alteromonas* type strains used in this study. Genomic sizes were obtained from NCBI, and genomic GC contents were calculated using SeqKit toolkit version 2.6.1 [37].

Type Strain	NCBI Assembly Accession Number	No. of Contigs	Size (Mbp)	GC Content (%)
*A*. *aestuariivivens* KCTC 52655^T^	GCA_003367475.1	35	3.85	50.5
*A*. *alba* 190^T^	GCA_002993365.1	221	5.16	48.7
*A*. *antoniana* MD_567^T^	GCA_019249295.1	90	4.95	48.9
*A*. *australica* H 17^T^	GCA_000730385.1	1	4.31	44.9
*A*. *chungwhensis* DSM 16280^T^	GCA_000378185.1	30	3.98	47.0
*A*. *confluentis* KCTC 42603^T^	GCA_001757105.1	46	4.86	48.0
*A*. *facilis* P0213^T^	GCA_002807665.1	23	3.91	44.7
*A*. *flava* P0211^T^	GCA_002807685.1	42	3.65	45.7
*A*. *genovensis* LMG 24078^T^	GCA_010500895.1	37	4.07	44.3
*A*. *gilva* chi3^T^	GCA_028595265.1	7	4.57	48.4
*A*. *gracilis* 9a2^T^	GCA_002993325.1	31	4.32	44.3
*A*. *halophila* KCTC 22164^T^	GCA_014651815.1	36	3.95	50.8
*A*. *hispanica* LMG 22958^T^	GCA_010500915.1	31	4.07	43.8
*A*. *iocasae* KX18D6^T^	GCA_006228385.1	2	4.16	47.3
*A*. *lipolytica* JW12^T^	GCA_001758465.1	21	5.02	48.4
*A*. *lipotrueae* MD_652^T^	GCA_019249215.1	44	4.75	43.7
*A*. *lipotrueiana* PS_109^T^	GCA_019249245.1	47	3.76	45.8
*A*. *lutimaris* DPSR-4^T^	GCA_005222225.1	1	4.12	49.8
*A*. *macleodii* ATCC 27126^T^	GCA_000172635.2	1	4.65	44.7
*A*. *mediterranea* DE^T^	GCA_000020585.3	1	4.48	44.9
*A*. *naphthalenivorans* SN2^T^	GCA_000213655.1	1	4.97	43.5
*A*. *oceani* S35^T^	GCA_003731635.1	90	4.99	48.7
*A*. *pelagimontana* 5.12^T^	GCA_002499975.2	1	4.31	46.1
*A*. *ponticola* MYP5^T^	GCA_012911815.1	22	3.60	46.1
*A*. *portus* HB161718^T^	GCA_005117025.1	32	4.54	44.1
*A*. *profundi* 345S023^T^	GCA_010500865.1	99	4.39	44.4
*A*. *sediminis* U0105^T^	GCA_003820355.1	14	3.96	45.3
*A*. *stellipolaris* LMG 21861^T^	GCA_001562115.1	2	4.90	43.5

**Table 2:** ANI and *is*DDH values between strain CYL-A6^T^ and other *Alteromonas* type strains.

Type Strain	ANI (%)	*is*DDH (%)
*A*. *aestuariivivens* KCTC 52655^T^	72.0	19.7
*A*. *alba* 190^T^	71.6	20.7
*A*. *antoniana* MD_567^T^	72.7	19.3
*A*. *australica* H 17^T^	71.0	19.4
*A*. *chungwhensis* DSM 16280^T^	70.5	18.9
*A*. *confluentis* KCTC 42603^T^	71.2	20.4
*A*. *facilis* P0213^T^	69.8	23.8
*A*. *flava* P0211^T^	69.0	20.5
*A*. *genovensis* LMG 24078^T^	70.8	19.9
*A*. *gilva* chi3^T^	70.5	20.1
*A*. *gracilis* 9a2^T^	71.2	20.6
*A*. *halophila* KCTC 22164^T^	73.7	19.0
*A*. *hispanica* LMG 22958^T^	70.7	19.8
*A*. *iocasae* KX18D6^T^	70.9	19.6
*A*. *lipolytica* JW12^T^	71.0	20.2
*A*. *lipotrueae* MD_652^T^	70.5	19.0
*A*. *lipotrueiana* PS_109^T^	70.6	18.8
*A*. *lutimaris* DPSR-4^T^	71.6	19.5
*A*. *macleodii* ATCC 27126^T^	71.4	20.8
*A*. *mediterranea* DE^T^	71.8	21.7
*A*. *naphthalenivorans* SN2^T^	71.0	19.5
*A*. *oceani* S35^T^	70.9	19.9
*A*. *pelagimontana* 5.12^T^	70.9	19.2
*A*. *ponticola* MYP5^T^	70.7	19.0
*A*. *portus* HB161718^T^	71.5	20.3
*A*. *profundi* 345S023^T^	70.5	19.5
*A*. *sediminis* U0105^T^	69.0	20.7
*A*. *stellipolaris* LMG 21861^T^	70.7	19.3

**Table 3:** Differential characteristics between strain CYL-A6^T^ and its reference strain, *A*. *halophila* KCTC 22164^T^. +, positive; −, negative. DNA G+C contents are calculated from genome sequence. *, data from Chen et al. (2009) [57].

Characteristic	Strain CYL-A6^T^	*A*. *halophila* KCTC 22164^T^
**Temperature for growth (°C):**		
Range	20–45	10–40 *
Optimum	37	25–30 *
**NaCl for growth (*w*/*v*, %):**		
Range	1.0–12.0	1.0–20.0 *
Optimum	3.5	5.0–10.0 *
**pH for growth:**		
Range	5.5–9.0	6.0–10.0 *
Optimum	7.0	7.5 *
**Hydrolysis of**		
Tween 40	−	+
**API ZYM:**		
α- and β-Galactosidase	−	+
β-Glucosidase	−	+
**API 20NE:**		
Hydrolysis of urea	+	−
β-Galactosidase	−	+
Nitrate reduction	+	−
**Utilization of:**		
D-Mannitol	−	+
**Acid production from:**		
D-Glucose and D-mannose	+	−
**DNA G+C content (%)**	51.8	50.8

**Table 4:** Fatty acid profiles of strain CYL-A6^T^ and its reference strain, *A*. *halophila* KCTC 22164^T^. Summed feature 2 contains iso-C_16:1_ I and/or C_14:0_ 3-OH; summed feature 3 contains C_16:1_*ω*6*c* and/or C_16:1_ *ω*7*c*; summed feature 7 contains cyclo-C_19:0_*ω*10*c* and/or C_19:1_*ω*6*c*; summed feature 8 contains C_18:1_*ω*6*c* and/or C_18:1_*ω*7*c*. Values are percentages of total fatty acids. Fatty acids lower than 0.2% in both strains were omitted.

Fatty Acids	Strain CYL-A6^T^	*A*. *halophila* KCTC 22164^T^
**Straight-chain**		
C_12:0_	2.0	3.5
C_14:0_	3.9	3.6
C_16:0_	22.6	23.7
C_17:0_	5.8	2.2
C_18:0_	2.1	1.2
**Branched-chain**		
iso-C_14:0_	0.6	0.2
iso-C_16:0_	1.3	0.6
iso-C_18:0_	0.6	0.2
**Unsaturated**		
C_15:1_*ω8c*	1.6	0.5
C_17:1_*ω8c*	6.6	2.9
**Hydroxy**		
C_10:0_ 3-OH	2.1	2.2
C_11:0_ 3-OH	1.6	0.5
C_12:0_ 3-OH	0.9	0.7
C_12:1_ 3-OH	1.4	2.6
**Summed feature 2**	3.7	3.2
**Summed feature 3**	20.3	28.0
**Summed feature 7**	1.6	1.3
**Summed feature 8**	12.4	17.6

**Table 5:** COG and KEGG annotations of gained OCs in nodes 28–37. COG categories are described as C (energy production and conversion), E (amino acid transport and metabolism), F (nucleotide transport and metabolism), G (carbohydrate transport and metabolism), H (coenzyme transport and metabolism), I (lipid transport and metabolism), K (transcription), M (cell wall/membrane/envelope biogenesis), N (cell motility), O (posttranslational modification, protein turnover, chaperones), P (inorganic ion transport and metabolism), Q (secondary metabolites biosynthesis, transport and catabolism), S (function unknown), and T (signal transduction mechanisms). OCs not assigned to KEGG databases or annotated as multiple KO numbers were omitted.

OCs	KO Number	Annotation	COG Category
OG0000020	K16264	*czcD*; cobalt-zinc-cadmium efflux system protein	P
OG0000050	K07814	cyclic di-GMP phosphodiesterase	T
OG0000059	K01271	*pepQ*; Xaa-Pro dipeptidase	E
OG0000079	K06222	*dkgB*; 2,5-Diketo-D-gluconate reductase B	S
OG0000175	K02083	*allC*; allantoate deiminase	E
OG0000214	K03307	*TC.SSS*; solute:Na^+^ symporter, SSS family	S
OG0000263	K03406	*mcp*; methyl-accepting chemotaxis protein	T
OG0000549	K07089	Uncharacterized protein	S
OG0000752	K02030	*ABC.PA.S*; polar amino acid transport system substrate-binding protein	ET
OG0001437	K03406	*mcp*; methyl-accepting chemotaxis protein	NT
OG0001606	K04065	*osmY*; hyperosmotically inducible periplasmic protein	S
OG0001699	K02429	*fucP*; MFS transporter, FHS family, L-fucose permease	G
OG0001868	K03885	*ndh*; NADH:quinone reductase (non-electrogenic)	C
OG0001942	K02014	*TC.FEV.OM*; iron complex outermembrane recepter protein	P
OG0002072	K01785	*galM*; aldose 1-epimerase	G
OG0002073	K09781	Uncharacterized protein	S
OG0002091	K02426	*sufE*; cysteine desulfuration protein	S
OG0002103	K13924	*cheBR*; two-component system, chemotaxis family, CheB/CheR fusion protein	T
OG0002106	K03314	*nhaB*; Na^+^:H^+^ antiporter, NhaB family	P
OG0002119	K03409	*cheX*; chemotaxis protein CheX	N
OG0002126	K03585	*mexA*; membrane fusion protein, multidrug efflux system	M
OG0002127	K18288	*ict-Y*; itaconate CoA-transferase	C
OG0002139	K09954	Uncharacterized protein	S
OG0002142	K00569	*tpmT*; thiopurine S-methyltransferase	Q
OG0002165	K08234	*yaeR*; glyoxylase I family protein	E
OG0002170	K06886	*glbN*; hemoglobin	S
OG0002173	K02529	*galR*; LacI family transcriptional regulator, galactose operon repressor	K
OG0002185	K01487	*guaD*; guanine deaminase	F
OG0002186	K13482	*xdhB*; xanthine dehydrogenase large subunit	F
OG0002187	K06901	*adeQ*; adenine/guanine/hypoxanthine permease	S
OG0002188	K20920	*vpsM*; polysaccharide biosynthesis protein VpsM	S
OG0002207	K06200	*cstA*; carbon starvation protein	T
OG0002216	K04761	*oxyR*; LysR family transcriptional regulator, hydrogen peroxide-inducible genes activator	K
OG0002218	K13481	*xdhA*; xanthine dehydrogenase small subunit	F
OG0002223	K01090	Protein phosphatase	IT
OG0002228	K09897	Uncharacterized protein	S
OG0002235	K03969	*pspA*; phage shock protein A	KT
OG0002238	K20444	*rfbC*; O-antigen biosynthesis protein	M
OG0002267	K09929	Uncharacterized protein	S
OG0002277	K03088	*rpoE*; RNA polymerase sigma-70 factor, ECF subfamily	K
OG0002278	K07107	*ybgC*; acyl-CoA thioester hydrolase	S
OG0002284	K08990	*ycjF*; putative membrane protein	S
OG0002285	K06889	Uncharacterized protein	S
OG0002291	K09689	*kpsT*; capsular polysaccharide transport system ATP-binding protein	GM
OG0002299	K16840	*hpxQ*; 2-oxo-4-hydroxy-4-carboxy-5-ureidoimidazoline decarboxylase	S
OG0002300	K00681	*ggt*; gamma-glutamyltranspeptidase/glutathione hydrolase	E
OG0002319	K16088	*fhuE*; outer-membrane receptor for ferric coprogen and ferric-Rhodotorulic acid	P
OG0002320	K06918	Uncharacterized protein	S
OG0002323	K01834	*gpmA*; 2,3-bisphosphoglycerate-dependent phosphoglycerate mutase	G
OG0002329	K07127	*hiuH*; 5-hydroxyisourate hydrolase	S
OG0002330	K07402	*xdhC*; xanthine dehydrogenase accessory factor	O
OG0002334	K11811	*arsH*; arsenical resistance protein ArsH	S
OG0002340	K14153	*thiDE*; hydroxymethylpyrimidine kinase/phosphomethylpyrimidine kinase/thiamine-phosphate diphosphorylase	H
OG0002351	K00262	*gdhA*; glutamate dehydrogenase (NADP+)	E
OG0002363	K02055	*ABC.SP.S*; putative spermidine/putrescine transport system substrate-binding protein	E
OG0002369	K00362	*nirB*; nitrite reductase (NADH) large subunit	C
OG0002370	K00372	*nasA*; assimilatory nitrate reductase catalytic subunit	C
OG0002371	K07023	*YGK1*; 5′-deoxynucleotidase	S
OG0002373	K10107	*kpsE*; capsular polysaccharide transport system permease protein	M
OG0002374	K12990	*rfbF*; rhamnosyltransferase	S
OG0002393	K03669	*mdoH*; membrane glycosyltransferase	M
OG0002394	K03670	*mdoG*; periplasmic glucans biosynthesis protein	P
OG0002397	K03149	*thiG*; thiazole synthase	H
OG0002403	K09688	*kpsM*; capsular polysaccharide transport system permease protein	GM
OG0002412	K06149	*uspA*; universal stress protein A	T
OG0002427	K00363	*nirD*; nitrite reductase (NADH) small subunit	P
OG0002430	K05782	*benE*; benzoate membrane transport protein	Q
OG0002442	K01759	*gloA*; lactoylglutathione lyase	E
OG0002448	K00847	*scrK*; fructokinase	G
OG0002483	K02303	*cobA*; uroporphyrin-III C-methyltransferase	H
OG0002496	K00344	*qor*; NADPH:quinone reductase	C
OG0002515	K03793	*PTR1*; pteridine reductase	IQ
OG0002516	K01104	Protein-tyrosine phosphatase	GM
OG0002523	K09797	Uncharacterized protein	S
OG0002534	K07238	*zupT*; zinc transporter, ZIP family	P
OG0002543	K01425	*glsA*; glutaminase	E
OG0002555	K15977	Putative oxidoreductase	S
OG0002594	K01083	3-Phytase	I
OG0002688	K16044	*iolW*; scyllo-inositol 2-dehydrogenase (NADP+)	S

## Data Availability

The original data presented in the study are openly available in a publicly accessible repository. The 16S rRNA gene and genome sequences of strain CYL-A6^T^ have been deposited in the GenBank database under accession numbers PQ035033 and JBFNPK000000000, respectively.

## Supplementary Materials

The following supporting information can be downloaded at: https://www.mdpi.com/article/10.3390/microorganisms13081888/s1, Figure S1: Neighbor-joining phylogenetic tree based on the 16S rRNA gene sequences showing phylogenetic relationships of strain CYL-A6^T^ and *Alteromonas* type strains. Bootstrap values are based on 1000 repetitions. Bar, 0.02 substitutions per nucleotide position. *Escherichia coli* NBRC 102203T (AB681728) was used as an outgroup. Figure S2: The nitrate reduction ability of strain CYL-A6^T^ and the reference strain *Alteromonas halophila* KCTC 22164^T^ was tested with nitrate broth and a nitrate reduction kit. Red reaction was determined as positive and was otherwise considered as negative. (a) Strain CYL-A6^T^; (b) *Alteromonas halophila* KCTC 22164^T^. Table S1: Genomic information and ecological gene annotations of high-quality *Alteromonas* genomes used in this study. + and − indicated presence and absence of genes, respectively. Table S2: Detailed 16S rRNA gene sequence identity and top hit type strains of 32 bacterial strains isolated and cultivated in this study. Table S3: Sequence identity results based on the 16S rRNA gene sequences of strain CYL-A6^T^ using the EzBioCloud web server. Table S4: Protein sequences and functional annotations of exclusive groups in the strain CYL-A6^T^. Table S5: Protein sequence of respiratory nitrate reductase in the strain CYL-A6^T^. Sequence identity results were obtained using BLAST searches against the Uniprot database. Table S6: Nitrate-reducing genes annotations based on the Uniprot database. Table S7: Gene sequence/protein sequence identities of the *narGHI* genes/NarGHI proteins in *Alteromonas* members. Upper and lower triangles indicated gene and protein sequence identities, respectively. Table S8: Protein sequences as well as COG and KEGG annotations of OCs that were exclusively present in the *Alteromonas* genomes encoding nitrate-reducing genes. Table S9: Orthologous cluster distributions in nodes 1 and 28–37.

## Abbreviations

The following abbreviations are used in this manuscript:

ANI	Average Nucleotide Identity
COG	Clusters of Orthologous Groups of proteins
GO	Gene Ontology
*is*DDH	in silico DNA–DNA Hybridization
KCTC	Korean Collection of Type Cultures
KEGG	Kyoto Encyclopedia of Genes and Genomes
MA	Marine Agar 2216
MCCC	Marine Culture Collection of China
Q-8	Ubiquinone-8
