# Supplementary material for: Alteromonas nitratireducens sp. nov., a Novel Nitrate-Reducing Bacterium Isolated from Marine Sediments, and the Evolution of Nitrate-Reducing Genes in the Genus Alteromonas
Source: Microorganisms. 2025 Aug 13;13(8):1888. doi: 10.3390/microorganisms13081888 (PMC12388587; doi:10.3390/microorganisms13081888)

Figure S1. Neighbor-Joining phylogenetic tree based on the 16S rRNA gene sequences showing phylogenetic relationships of strain CYL-A6^T and *Alteromonas* type strains. Bootstrap values were based on 1000 repetitions. Bar, 0.02 substitutions per nucleotide position. *Escherichia coli* NBRC 102203^T (AB681728) was used as an outgroup.

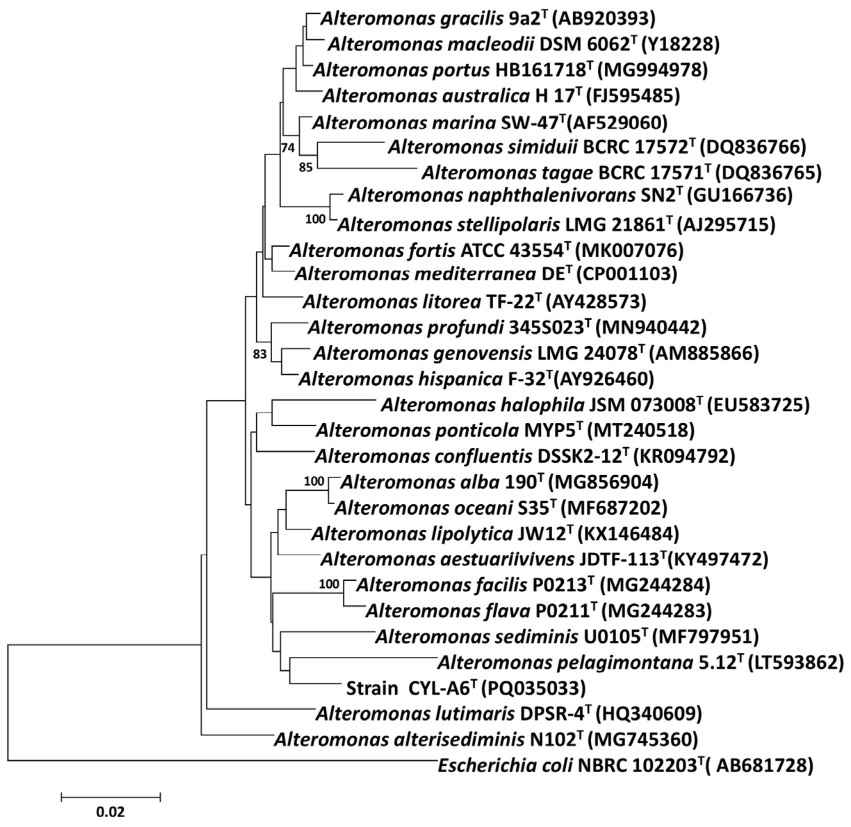

Figure S2. The nitrate reduction ability of strain CYL-A6^{T} and the reference strain *Alteromonas halophila* KCTC 22164^{T} was tested with nitrate broth and nitrate reduction kit. Red reaction was determined as positive, and otherwise was considered as negative. (a) Strain CYL-A6^{T}; (b) *Alteromonas halophila* KCTC 22164^{T}.

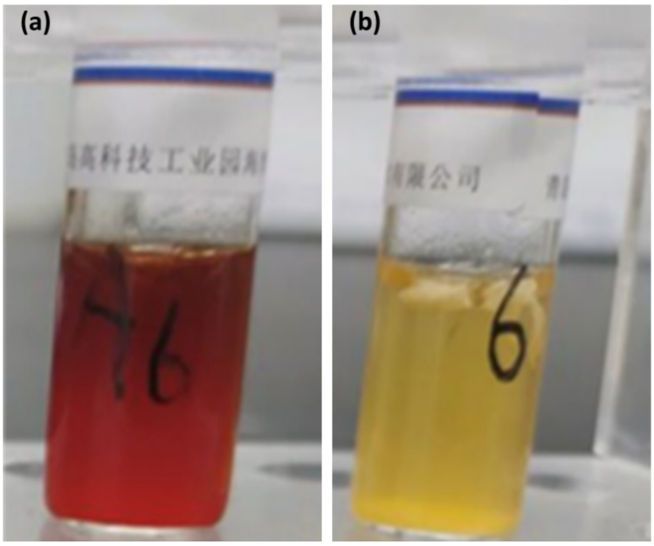

Supplement: Supplementary file 1 [file microorganisms-13-01888-s001.zip › Supplementary Figures.pdf]
